# DNA methylation changes between relapse and remission of minimal change nephrotic syndrome

**DOI:** 10.1007/s00467-012-2248-z

**Published:** 2012-08-02

**Authors:** Yasuko Kobayashi, Akira Aizawa, Takumi Takizawa, Chikage Yoshizawa, Hiromi Horiguchi, Yuka Ikeuchi, Satoko Kakegawa, Toshio Watanabe, Kenichi Maruyama, Akihiro Morikawa, Izuho Hatada, Hirokazu Arakawa

**Affiliations:** 1 Department of Pediatrics, Gunma University Graduate School of Medicine, 3-39-22 Showa-machi, Maebashi, Gunma 371-8511 Japan; 2 Department of Pediatrics, Gunma Chuo General Hospital, Maebashi, Gunma Japan; 3 Gunma Children’s Medical Center, Hokkitsumura, Seta-gun, Gunma Japan; 4 Kitakanto Allergy Institute, Midorishi, Gunma Japan; 5 Laboratory of Genome Science, Biosignal Genome Resource Center, Gunma University Institute for Molecular and Cellular Regulation, Maebashi, Gunma Japan

**Keywords:** DNA methylation, Nephrotic syndrome, Monocytes, Naive T helper cells, Microarray-based integrated analysis of methylation by isoschizomers (MIAMI) method, Genome-wide, Children

## Abstract

**Background:**

DNA methylation of gene promoters is associated with transcriptional inactivation. Changes in DNA methylation can lead to differences in gene expression levels and thereby influence disease development. We hypothesized that epigenetics underlies the pathogenesis of minimal change nephrotic syndrome (MCNS).

**Methods:**

Genome-wide DNA methylation changes between relapse and remission in monocytes (*n* = 6) and naive T helper cells (Th0s) (*n* = 4) isolated from patients with MCNS were investigated using the microarray-based integrated analysis of methylation by isochizomers (MIAMI) method. We confirmed the MIAMI results using bisulfite-pyrosequencing analysis. Expression analysis was performed using quantitative real-time PCR.

**Results:**

Three gene loci (*GATA2*, *PBX4*, and *NYX*) were significantly less methylated in Th0s during relapse than in remission, compared to none in monocytes. In addition, the distance distribution from the regression line of all probes in MIAMI was significantly different between monocytes and Th0s. The mRNA levels of the three genes in Th0s were not significantly different between relapse and remission.

**Conclusions:**

Our results demonstrate that the change in DNA methylation patterns from remission to relapse in MCNS occurs predominantly in Th0s rather than in monocytes and suggest that epigenetic regulation in Th0s underlies the pathogenesis of MCNS.

**Electronic supplementary material:**

The online version of this article (doi:10.1007/s00467-012-2248-z) contains supplementary material, which is available to authorized users.

## Introduction

Epigenetics is the study of mitotically heritable changes in gene expression that occur without direct DNA sequence alterations. DNA methylation, one of the principal epigenetic mechanisms in mammals, involves the covalent addition of a methyl group to a cytosine residue that is followed by a guanine (CpG) [1]. DNA methylation regulates gene expression and is essential for differentiation, embryonic development [2], genomic imprinting [3], and X-chromosome inactivation [4]. DNA methylation within the promoter region of a gene is commonly associated with transcriptional inactivation, whereas demethylation contributes to transcriptional activation. Changes in the DNA methylation profile can also lead to differences in gene expression patterns and thereby influence the development of diseases, such as cancer [5].

Minimal change nephrotic syndrome (MCNS) is the most common cause of nephrotic syndrome in children and is characterized by massive proteinuria and hypoalbuminemia in a relapse/remission course without histological evidence of immune-mediated inflammatory damage. These manifestations are typically reversible with the use of corticosteroid therapy. Although the pathogenesis of MCNS remains to be elucidated, immunological disruption has been implicated in this disease [6] as T cell-derived vascular permeability factors have been shown to be responsible for alterations in glomerular permeability [7–9]. The incidence of MCNS in childhood is twofold higher in boys, with a prevalence that is inversely proportional to age, and recurrent relapse tends to lessen after adolescence [10, 11]. Since the characteristic features of MCNS include (1) a recurrent relapse/remission course, (2) gender preference, (3) age preference of onset and relapse, and (4) steroid response in most patients, a genetic defect cannot explain the pathogenesis of this disease; however, epigenetic alterations may occur without a direct change in the genetic sequence. DNA methylation changes with age and environmental factors, even in the same individual, and is involved with X-chromosome inactivation.

Audard et al. reported that NFRKB (nuclear factor related to kappaB binding protein) was highly expressed in the nuclear compartment during relapse and that NFRKB promotes hypomethylation of genomic DNA, suggesting epigenetic involvement in the pathogenesis of MCNS [12]. The epigenotype is influenced by the environment and alters the regulation of gene expression, leading Elie et al. to suggest a probable impact of epigenetic modifications in infected cells since MCNS relapses are frequently triggered by external or internal environmental factors, including viral infection [13]. Zhang et al. reported significant differences in histone H3 lysine 4 tri-methylation of peripheral blood mononuclear cells (PBMCs) from adult patients with MCNS compared with those from healthy subjects. Their results indicate that alterations in epigenotype are associated with the pathogenesis of MCNS [14].

The aim of this study is to elucidate whether the DNA methylation profile changes between relapse and remission in MCNS cases and whether this process is immune-competent cell-type-specific. Ultimately, we wished to determine whether epigenetics underlies the pathogenesis of MCNS.

## Patients and methods

### Patients

Samples for microarray-based integrated analysis of methylation by isochizomers (MIAMI) analysis were obtained from six male patients with MCNS (Table 1), while samples for quantitative real-time PCR (qRT-PCR) were obtained from an additional seven patients with MCNS (3 boys, 4 girls) (Table 2) at relapse and also following complete remission. All patients were diagnosed according to the criteria of the International Study of Kidney Disease in Children [15] and had developed nephrotic syndrome prior to 16 years of age. Informed consent was obtained from the parents of each child and from older children/adolescents as necessary. This study was approved by the Ethics Committee of Gunma University Graduate School of Medicine, Japan (Receipt Number 89).

### Cell separation

We used monocytes, which are precursors of the antigen-presenting cells derived from the myeloid cell series, and naive T helper cells (Th0s), which are derived from the lymphoid system, as material for the analyses. PBMCs were isolated from 20-mL samples of anti-coagulated blood that had been obtained by gradient separation using the Lymphprep™ Tube system (Axis-Shield PoC AS, Oslo, Norway). Monocytes and Th0s were separated from PBMCs that had been magnetically labeled with CD14, CD4, and CD45RO microBeads (Miltenyi Biotec) using an autoMACS Pro Separator (Miltenyi Biotec, Bergisch Gladbach, Germany) . To obtain monocytes, the CD14-positive (CD14^+^) fraction was collected as monocytes, whereas the CD14-negative, CD4-positive, and CD45RO-negative (CD14^-^CD4^+^CD45RO^-^) fractions were collected as Th0s. Flow cytometric analysis with a MACSQuant® Analyzer (Miltenyi Biotec) revealed that the precision of cell separation was 96.2 % for CD14^+^ cells and 94.46 % for CD4-positive and CD45RA-positive cells as a CD45RO-negative fraction.

CD14^+^ monocytes were obtained from six patients, while CD14^-^CD4^+^CD45RO^-^ Th0 cells were also obtained from four of these patients (Table 1) for the MIAMI analysis both at relapse and following complete remission. Genomic DNA (gDNA) was extracted from these cells as previously described [16]. Consistent amounts of extracted gDNA (300 ng from monocytes and 250 ng from Th0s) from each selected cell type were pooled and used for subsequent DNA methylation analysis using MIAMI in order to exclude the influences of epigenetic differences among the cell types, to identify specific changes due to clinical courses of MCNS, and to reduce the noise caused by individual differences. Total RNA was extracted from Th0s purified from seven other patients with MCNS (Table 2) using a ToTALLY RNA™ kit (Ambion, Austin, TX). Complementary DNA (cDNA) was synthesized from total RNA of each Th0s sample using the High Capacity RNA-to-cDNA Master Mix (Applied Biosystems, Foster City, CA) for the qRT-PCR.

### MIAMI analysis

The MIAMI method, which provides high-throughput global analysis of DNA methylation, was performed as described previously using 1.8 and 1.0 μg of pooled gDNA isolated from the monocytes of six patients and from the Th0s of four patients, respectively [17, 18]. Briefly, this technique utilizes isoschizomers (*Hpa*II and *Msp*I) that recognize the same recognition site (CCGG). Pooled gDNA was digested with *Hpa*II, a methylation-sensitive restriction enzyme that cleaves only unmethylated DNA, and then adapter-ligated and amplified by PCR with primers designed against the adapter sequences. The samples were then further digested with *Msp*I, a methylation-insensitive enzyme that digests CCGG sites irrespective of their methylation status, and amplified again with the same set of primers (*Hpa*II–*Msp*I treatment). The second treatment with *Msp*I yields amplicons from unmethylated DNA fragments only. Hence, only *Hpa*II cleavable unmethylated DNA fragments are amplified, and these can then be quantified based on their respective fluorescence intensity by microarray analysis. The amplified products were then labeled with Cy3 (remission samples) or Cy5 (relapse samples) and co-hybridized to a microarray spotted with 38,172 sixty-mer oligonucleotides covering the vicinity of the transcription start sites (TSSs) of 14,978 genes.

Following hybridization, the microarray was scanned, and the obtained fluorescence intensities were quantified and normalized. The same pooled gDNA samples were treated first with *Msp*I instead of *Hpa*II (*Msp*I–*Msp*I treatment) and analyzed on a duplicate array to correct for false-positives caused by single nucleotide polymorphisms or incomplete digestion.

### Bisulfite-pyrosequencing analysis

In the bisulfite-pyrosequencing method, unmethylated cytosine residues are converted into uracil, whereas methylated cytosines remain unchanged. Analysis of the methylation status in this manner exploits the quantitative nature of pyrosequencing by reporting the ratio of cytosine to thymine at each analyzed CpG site, which reflects the proportion of methylated DNA. This analysis was performed using pooled samples in accordance with established protocols. Briefly, gDNA extracts from patients were digested with *Eco*RI (Takara Bio, Otsu, Japan) and subjected to bisulfite treatment using the EZ DNA Methylation-Gold Kit (Zymo Research, Orange, CA). The analyzed CpG sites were in the closest *Hpa*II recognition sites at the 5′- and 3′- ends of the probes designed in the TSSs vicinity of *GATA2* (A_17_P02574948), *PBX4* (A_17_P10909964), and *NYX* (A_17_P11717994) used in the MIAMI analysis. Amplification and sequencing primers for pyrosequencing were designed with PyroMark Assay Design software ver. 2.0 (Qiagen, Venlo, the Netherlands) (Electronic Supplementary Material Table 1).

The targeted DNA segments were amplified using a hot start protocol with a touchdown PCR system (Veriti™ Thermal Cycler; Applied Biosystems), and the strand serving as the pyrosequencing template was biotinylated. Following denaturation, the biotinylated single-stranded PCR amplicons were isolated and allowed to hybridize with a sequencing primer. Pyrosequencing (PyroMark Gold Q96 Reagents; Qiagen) was then performed using the PyroMark Q24 system (Qiagen) according to the manufacturer’s protocol. The sequencing assay was validated using an internal control (a non-CpG cytosine within the target methylation sequence region).

### Quantitative real-time PCR

The qRT-PCR analysis was performed using the TaqMan PCR method with a 7900HT Fast Real-Time PCR System (Applied Biosystems) with cDNA from Th0s separated from the other patients with MCNS at relapse and subsequent remission. The three genes previously analyzed using MIAMI, namely, *GATA2* (Hs00231119_m1), *PBX4* (Hs00257935_m1), and *NYX* (Hs00360869_m1), were assayed. Each gene was assayed four times for each sample. Aliquots of cDNA equivalent to 300 ng of total RNA were used in the RT-PCR reactions. *GAPDH* (Hs99999905_m1) was used as an endogenous control for normalizing the RNA concentrations. Differences in the C_T_ values between the tested genes and endogenous control (ΔC_T_) were calculated and used for subsequent statistical analyses.

### Statistical analysis

**Fig. 1:**
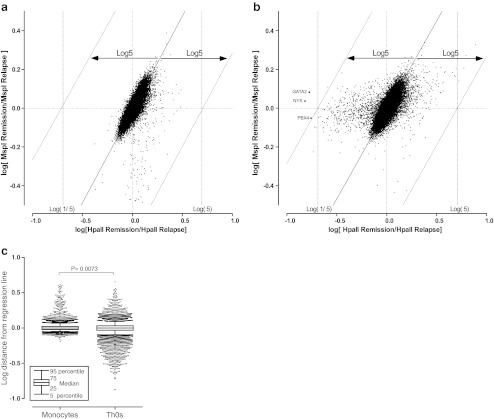
**a**, **b** Scatter plots of the signals obtained for each probe in monocytes (**a**) and naive T helper cells (**b**). Log [(*Hpa*II intensity) Remission/(*Hpa*II intensity) Relapse] values are plotted on the *x*-axis and log[(*Msp*I intensity) Remission/(*Msp*I intensity) Relapse] values are plotted on the *y*-axis. The threshold values are determined at log5 of the horizontal distance from the center of the mass and at log5 of the horizontal distance to the regression line of the plots in accordance with the original MIAMI method of Hatada et al. [17, 18]. *Points* located on the *right side* and *beyond* the distance of these *lines* are judged to be more highly methylated; those located to the *left* are judged to be significantly less methylated in the relapse samples compared with the remission samples. The three gene probes were found to be less methylated in relapse samples than in remission samples in naive T helper cells (**b**), whereas no significant signal was detected in monocytes (**a**). **c** Distance distributions of all probes are from the regression line. Each *dot* indicates the log distance of the indicated probes plotted out to the 90th percentile of signals from the regression line. These distributions were significantly different (*P* = 0.0073) between monocytes and naive T helper cells (*Th0s*)

Statistical analyses of the distance distributions from the MIAMI regression line for each of the probes used in the assays was performed using a non-parametric Mann–Whitney *U* test (Fig. 1). Comparisons between the expression levels for each group were analyzed using the Wilcoxon matched-pairs signed-rank test (Fig. 4a, b). All statistical analyses were performed using GraphPad PRISM5 software (GraphPad Software, La Jolla, CA) with the significance level set at *P* < 0.05.

## Results

### Patients for DNA methylation analysis

We first investigated whether any genome-wide changes in DNA methylation occurred between relapse and remission in the patients with MCNS. The mean total follow-up period until June 2011 was 109.5 months (range 35–188 months). All patients were steroid responsive and frequent relapsers within their total clinical course. Renal biopsy was performed during the follow-up period in four patients who had been administered cyclosporine because of their frequent relapses in order to histologically evaluate the side effects of cyclosporine. The histological findings were consistent with the diagnosis of MCNS. The ages at sampling for relapse and the total number of relapses until samples were collected are listed in Tables 1 and 2. The mean age at relapse sampling was 13 years and 5 months (range 6 years–19 years and 10 months), and the mean number of relapses at the time of sampling was six (range 3–13 relapses). The mean sampling interval from the relapse to the subsequent remission was 25 (range 5–71) weeks. The therapeutic conditions were similar at the time of relapse and remission sampling in all subjects except for patients 2 and 6, who received a corticosteroid (patients 2 and 6) and immunosuppressant (patient 2) at remission (Tables 1, 2).

**Table 1:** Characteristics of patients with minimal change nephrotic syndrome at sampling whose DNA was isolated for methylation analysis

Patient no.	Sex	Age at onset	Total follow-up period^a^ (months)	Frequent relapser	Biopsy	Sampling age at relapse	Relapse times at sampling	Intervals^b^ (weeks)	PSL at relapse (mg/day)	PLS at remission (mg/day)	IS at relapse (mg/day)	IS at remission (mg/day)	CD14+ (*n* = 6)	RO− (*n* = 4)
1	M	10y4m	78	Yes	MC	13y0m	3rd	5	40	10	0	0	Taken	Taken
2	M	5y4m	35	Yes	MC	6y0m	3rd	16	0	35	0	CyA90	Taken	Taken
3	M	5y11m	186	Yes	MC	18y6m	9th	23	10	15	0	0	Taken	Taken
4	M	7y3m	188	Yes	MC	19y10m	13th	23	60	5	0	0	Taken	Taken
5	M	9y9m	109	Yes	ND	11y7m	4th	12	0	0	CyA180	CyA160	Taken	NT
6	M	11y11m	61	Yes	ND	12y5m	4th	71	0	20	MZV125	MZV 75	Taken	NT
Mean		8y4m	109.5			13y5m	6th	25						

PSL*,* prednisolone IS*,* immunosuppressant; M, Male; y, years; m, months; MC, minimal change; ND, not done; CyA*,* cyclosporin A; MZB*,* mizoribine; NT*,* not taken

^a^Follow-up period till June 2011

^b^Intervals of sampling between relapse and remission

**Table 2:** Characteristics of patients with minimal change nephrotic syndrome at sampling who underwent gene expression analysis

Patient no.	Sex	Age at onset	Total follow-up period^a^ (months)	Frequent relapser	Biopsy	Sampling age at relapse	Relapse times at sampling (n)	Intervals^b^ (weeks)	PSL at relapse (mg/day)	PLS at remission (mg/day)	IS at relapse (mg/day)	IS at remission (mg/day)
7	M	8y4m	121	Yes	MC	11y11m	8	1	10	60	MZV150	MZV150
8	M	10y4m	78	Yes	MC	13y8m	5	2	0	0	0	0
9	M	2y5m	154	Yes	MC	17y9m	20	3	60	40	MZV150	MZV150
10	F	4y3m	44	No	ND	6y2m	2	26	0	0	0	0
11	F	4y10m	130	Yes	ND	15y0m	8	1	0	30	0	0
12	F	10y3m	36	Yes	ND	14y0m	4	13	0	20ADT	CyA50	CyA50
13	F	4y7m	37	Yes	MC	6y7m	7	8	0	0	CyA80	CyA80
Mean		6y5m	85.7			12y1m	7.7	7.7				

F, Female

^a^Follow-up period till June 2011

^b^Intervals of sampling between relapse and remission

### MIAMI analysis

The scatter plots of the signals from each probe in monocytes and Th0s are shown in Fig. 1a and b. The values for log [(*Hpa*II intensity) remission/(*Hpa*II intensity) relapse] are plotted on the x-axis, which represents the relative methylation changes upon relapse compared with those at remission. The values for the log [(*Msp*I intensity) remission/(*Msp*I intensity) relapse] are plotted on the y-axis and represent the control of the enzyme effects at sample digestion. The threshold values were determined according to the original MIAMI method described by Hatada et al [17, 18]. Three probes were found to be less methylated in the relapse Th0s samples compared with those taken at remission, whereas none of the probes had a significant detectable signal in monocytes (Fig. 1a and b). The distance distributions of all probes from the regression line were significantly different (*P* = 0.0073) between monocytes and Th0s (Fig. 1c). These results indicate that the DNA methylation status undergoes changes between relapse and remission in Th0s from MCNS patients and that the regulation of DNA methylation differs between monocytes and Th0s.

**Fig. 2:**
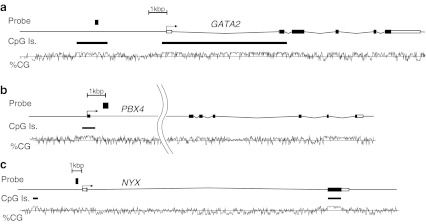
Gene maps of *GATA2* (**a**), *PBX4* (**b**), and *NYX* (**c**), including the position of the probes used in the MIAMI analysis. The number of CpG (covalent addition of a methyl group to a cytosine residue followed by a guanine) islands and the CG percentages throughout the genomic regions of these genes are also indicated. GATA binding protein 2 (*GATA2*) maps to chromosome (Ch) 3 q21.3, pre-B-cell leukemia homeobox 4 (*PBX4*) maps to Ch19 p12, and nyctalopin (*NYX*) maps to Ch X p11.4. *Filled squares* indicate the position of the probes with significant changes to the methylation status in Th0s. Probe A_17_P02574948 for *GATA2* recognizes the region 3.6 kb upstream of the transcription start site of this gene, which is within a CpG island. The probe A_17_P10909964 sequence in *PBX4* is located within the first intron of this gene, and probe A_17_P11717994 for *NYX* maps to the region just upstream of the transcription start site

The three genes detected in Th0s were GATA binding protein 2 (*GATA2*) at chromosome (Ch) 3 q21.3, pre-B-cell leukemia homeobox 4 (*PBX4*) at Ch 19 p12, and nyctalopin (*NYX*) at Ch X p11.4. The locations of the probes used to detect these three genes are shown in Fig. 2. The remission-to-relapse signal intensity ratios measured by MIAMI for the three probes following *Hpa*II treatment are shown in Table 3 and indicate the unmethylated intensity ratio between relapse and remission.

**Table 3:** Signal intensity ratio in *Hpa*II-treated samples for the indicated gene probes

Cell type	Gene	Probe	A^a^	Assessed^b^
Naive T cells	*GATA2*	A_17_P02574948	0.167722527	−1
	*PBX4*	A_17_P10909964	0.174326395	−1
	*NYX*	A_17_P11717994	0.151913072	−1
Monocytes	*GATA2*	A_17_P02574948	2.523381325	0

^a^Signal intensity ratio calculated by [(*Hpa*II intensity) Remission/(*Hpa*II intensity) Relapse] indicating an unmethylated intensity ratio for remission vs. relapse

^b^−1, Signal intensity ratio at this probe is located further left than the threshold lines indicating significantly lower methylation in the relapse samples than in the remission samples; 0, signal intensity ratio at this probe is located within the threshold lines

### Bisulfite-pyrosequencing analysis

**Fig. 3:**
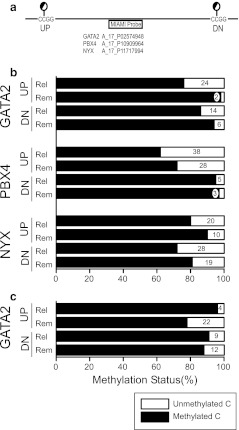
DNA methylation ratio analysis by bisulfite-pyrosequencing. To validate the MIAMI data, methylation ratios for CpGs in the closest CCGG *Hpa*II recognition sites on both the 5′ (*UP*) and 3′ (*DN*) side of the probe sequences were determined using bisulfite-pyrosequencing (**a**). These ratios were lower in minimal change nephrotic syndrome (MCNS) relapse (*Rel*) samples than in remission (*Rem*) samples at all sites for the three probes in Th0s (**b**). The methylation ratios for the *GATA2* probe A_17_P02574948 in monocytes (**c**) were higher in Rel than in Rem at both sides of the probe. The methylation pattern at every CpG site determined using bisulfite-pyrosequencing accorded well with the MIAMI results (Table 3), which indicate an unmethylated intensity ratio between relapse and remission

To confirm the results of the MIAMI analysis, we directly analyzed the methylation ratio using bisulfite-pyrosequencing for the CpGs that were in the closest CCGG *Hpa*II recognition sites on either side of the recognition sequences of all 3 probes tested in MIAMI analysis (Fig. 3a). At all sites in this analysis, the methylation ratios in Th0s were found to be lower at relapse than in remission (Fig. 3b). The methylation ratios for the *GATA2* probe A_17_P02574948 in monocytes were also tested and found to be higher at relapse than in remission on both sides of the probe (Fig. 3c). The methylation status at every CpG site determined by bisulfite-pyrosequencing analysis accorded well with the earlier MIAMI data (Table 3).

### qRT-PCR analysis

**Fig. 4:**
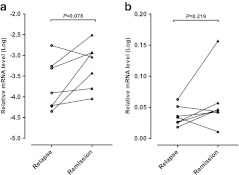
Expression of *GATA2* and *PBX4* genes in Th0s from patients with minimal change nephrotic syndrome at relapse and in remission. Nyctalopin (*NYX*) was not detected in Th0s. **a** *GATA2* expression was low and did not change significantly (*P* = 0.0781) between relapse and remission. **b** *PBX4* expression did not differ significantly (*P* = 0. 2188) between relapse and remission

According to the MIAMI analysis results, expression of the 3 genes under examination was determined in Th0s from the other patients with MCNS (Table 2) at relapse and subsequent remission using qRT-PCR (Fig. 4). *GATA2* expression was low and did not change significantly between relapse and remission (*P* = 0.0781; Fig. 4a). *PBX4* expression was also detectable but did not change significantly (*P* = 0. 2188; Fig. 4b). *NYX* was not amplified with the TaqMan probe.

## Discussion

A number of different approaches have been adopted in attempts to elucidate the mechanisms underlying alterations in the glomerular capillary permeability in MCNS. These have included studies of the capillary loop membrane, including the podocytes [19–23], searches for secretory factors which may change the permeability of the membrane [4, 24, 25], and investigations of immunocytes as a source of these factors [26, 27]. In the study reported here, we focused on immunocytes and their epigenetic regulation. We specifically isolated and studied Th0s and monocytes rather than using PBMCs, which usually include a wide range of cell types at different differentiation stages, as the latter cells would likely have a distinct epigenotype that could change even through the normal differentiation of Th0s to T helper cells, subset 2 (Th2) [28, 29]. We targeted naive or precursor cells in our search for possible predisposing causes of MCNS to exclude other influences on disease activity, such as Th2 activation, which has been reported in MCNS [30–33].

Differences in epigenetic patterns have been reported to occur even in genetically identical twins, which may be due to the influence of environmental factors [34]. In our experiments, it was possible to eliminate the effects of environmental or aging factors, which can impact on epigenotype variation among individual subjects, by comparing the DNA methylation differences in samples from the same individual obtained within short intervals (Tables 1, 2). However, it was impossible to exclude the effects of treatment with corticosteroids and cyclosporine A, which one and four patients received, respectively, although we could not find clear evidence of the impact of those medicines on the regulation of DNA methylation in immunocytes.

*GATA2* is a member of the GATA family of zinc-finger transcription factors (TFs), which play an essential role in the hematopoietic and endocrine systems (http://www.ncbi.nlm.nih.gov/pubmed?term=gata2). *PBX4* is a homeodomain protein similar to a TF that is involved in translocations in pre-B-cell leukemia (http://www.ncbi.nlm.nih.gov/pubmed?term=PBX4). *NYX* is a member of the small leucine-rich proteoglycan family of proteins in which defects can cause congenital stationary night blindness type 1, a rare inherited retinal disorder (http://www.ncbi.nlm.nih.gov/pubmed?term=nyx). Our finding that the DNA methylation ratios were lower in MCNS relapse samples within the promoter regions of these genes suggests that their expression might be higher at relapse than in remission. However, the expression of *GATA2* and *PBX4* were not higher at relapse in Th0s. One possible explanation for this result is that these two genes are in a primed state for transcription but are not necessarily activated in naive T cells. Changes in DNA methylation may precede changes in gene expression and influence the differentiation of Th0s into effector Th cells and/or influence gene expression after differentiation, which in turn affects the immunological and clinical states of MCNS.

Our findings that DNA methylation levels differ between the tested cell types—one from the myeloid series of cells and the other from the lymphoid system—indicates that it is necessary to separate the different types of immunocytes to investigate the epigenetic regulation of associated diseases, even though they are included in the general PBMC population. Although further analysis is required to determine candidate genes for MCNS, we conclude from our results data that the regulation of DNA methylation in Th0s, but not in monocytes, differs significantly between relapse and remission in affected patients and that epigenetic regulation in Th0s underlies the pathogenesis of MCNS, whose disturbances have been implicated in the development of the disease.

## Electronic supplementary material

Below is the link to the electronic supplementary material.

ESM 1 (DOCX 19 kb)
