# Supplementary material for: DNA methylation changes between relapse and remission of minimal change nephrotic syndrome
Source: Pediatr Nephrol. 2012 Aug 2;27(12):2233–41. doi: 10.1007/s00467-012-2248-z (PMC3491205; doi:10.1007/s00467-012-2248-z)
Supplement: Supplementary file 1 — (DOCX 19 kb) [file 467_2012_2248_MOESM1_ESM.docx]

Supplemental Table 1. Primers used for pyrosequencing

|  | Forward primer | Reverse primer-Biotin tag | Sequence primer |
| --- | --- | --- | --- |
| GATA2-1 | GATGTAATTGTGAGGGTGTGTTAAATAAG | CACCCCCTCCTATATAAACCCTCCAC | ATGGTAGGAGTTTGTGTTG |
| GATA2-2 | ATTGTGTTTGTTTTGGAGGTGGTTG | ACACACAACACATCCACCCTAAT | GGTTGTTTAGGGGAGTA |
| PBX4-1 | ATGTATTAGAGAGTAGGGATTATGTTAGTA | AAACCTATTTTCTTTTTAATAAATTCAACA | GAGAGTAGGGATTATGTTAGTATT |
| PBX4-2 | TATTTGTTTTGGAAATTTTTTATTTATAGG | ACCTCCCTAAATACTAAAATTAC | GTTTTGGAAATTTTTTATTTATAGGT |
| NYX-1 | GGAGGTTGTAGTGAGTTAAGATTGTG | TTCTAAAACCCACCAAAACTCAATT | GGTATTGTTGTTGGTTTAT |
| NYX-2 | GGGAATTTTTTGTTTTTTTGTAGTGGAA | CCACCCAAACAACATTATCAACATTTT | ATTTTAAAGAAAGGAAATTAGTAG |

-1 primers incorporate the closest *Hpa*II site from the 5′- end of the probe

-2 primers incorporate the closest *Hpa*II site from the 3′-end of the probe
